# Reading sentences of uniform word length – II: Very rapid adaptation of the preferred saccade length

**DOI:** 10.3758/s13423-018-1473-2

**Published:** 2018-04-25

**Authors:** Michael G. Cutter, Denis Drieghe, Simon P. Liversedge

**Affiliations:** 1 Department of Psychology, University of Edinburgh, 7 George Square, Edinburgh, EH8 9JZ UK; 2 School of Psychology, University of Southampton, Southampton, UK; 3 School of Psychology, University of Central Lancashire, Preston, UK

**Keywords:** Eye movements, Reading, Saccadic targeting, Systematic range error, Preferred saccade length

## Abstract

In the current study we investigated whether readers adjust their preferred saccade length (PSL) during reading on a trial-by-trial basis. The PSL refers to the distance between a saccade launch site and saccade target (i.e., the word center during reading) when participants neither undershoot nor overshoot this target (McConkie, Kerr, Reddix, & Zola in Vision Research, 28, 1107-1118, 1988). The tendency for saccades longer or shorter than the PSL to under or overshoot their target is referred to as the range error. Recent research by Cutter, Drieghe, and Liversedge (Journal of Experimental Psychology: Human Perception and Performance, 2017) has shown that the PSL changes to be shorter when readers are presented with 30 consecutive sentences exclusively made of three-letter words, and longer when presented with 30 consecutive sentences exclusively made of five-letter words. We replicated and extended this work by this time presenting participants with these uniform sentences in an unblocked design. We found that adaptation still occurred across different sentence types despite participants only having one trial to adapt. Our analyses suggested that this effect was driven by the length of the words readers were making saccades away from, rather than the length of the words in the rest of the sentence. We propose an account of the range error in which readers use parafoveal word length information to estimate the length of a saccade between the center of two parafoveal words (termed the Centre-Based Saccade Length) prior to landing on the first of these words.

## Introduction

During reading saccadic eye movements allow readers to extract high acuity visual information from multiple successive points in a sentence (Rayner, 1998). In English, saccades are argued to be typically targeted towards the center of an upcoming word, thus maximizing the number of a word’s letters that fall in high acuity vision, thereby increasing processing efficiency. However, fixation positions are normally distributed across the word with a mean landing position slightly to the left of the word center, due to several variables. One of the most important determinants of fixation landing positions is the distance between the previous fixation, and the center of the word that the eyes are moving towards. McConkie, Kerr, Reddix, and Zola (1988) examined the distribution of initial fixation positions within a word as a function of the distance of the site from which a saccade had been launched, finding that the eye tended to land in the center of a word when the saccade was launched from seven characters away from that word’s center. For every character that the launch site was further, or closer, to the word center, the mean landing position shifted 0.40 characters towards the start or end of the word, respectively. McConkie et al. concluded that a systematic range error exists within saccadic targeting, comprised of two fixed components. The first component is the preferred saccade length (PSL), defined as the intended saccade length that is not biased to either under- or overshoot its intended target (i.e., the word center). In English this is considered to be seven characters. The second component is the level of error predicted to occur for each character of deviation between (a) the PSL, and (b) the distance between the launch site and the intended saccade landing position, with this being 0.40 characters per character of deviation. The total error level is computed as the difference between the PSL and the distance between the current launch site and the word center, multiplied by a factor of 0.40 characters. Note also that McConkie et al. observed random motor error in saccadic targeting as well, such that while there is a modal landing position for any given launch site, fixation locations are normally distributed around this average.

**Table 1.** Linear mixed-model analyses for fixation landing position data

	Full analysis			Reduced analysis		
	b	SE	t	b	SE	t
Intercept	0.002	0.258	0.01	0.672	0.622	1.08
Uniformity	**-1.207**	**0.500**	**-2.42**	0.717	1.234	0.58
Length	**0.567**	**0.063**	**9.02**	**0.560**	**0.140**	**4.01**
Launch site	**-0.170**	**0.040**	**-4.26**	**-0.414**	**0.135**	**-3.08**
Uniformity × Length	**0.313**	**0.122**	**2.57**	-0.178	0.277	-0.64
Uniformity × Launch site	0.026	0.076	0.35	-0.174	0.267	-0.65
Length × Launch site	**-0.060**	**0.009**	**-6.48**	-0.028	0.029	-0.97
Uniformity × Length × Launch site	0.009	0.018	-0.50	0.042	0.058	0.72

*Note.* Significant terms are presented in bold. Full analysis refers to the LMM run on a full dataset, and reduced analysis refers to the analysis in which we only examined instances when participants moved between two words of the same length in the non-uniform sentences and between adjacent words in the uniform sentences

**Fig. 1:**
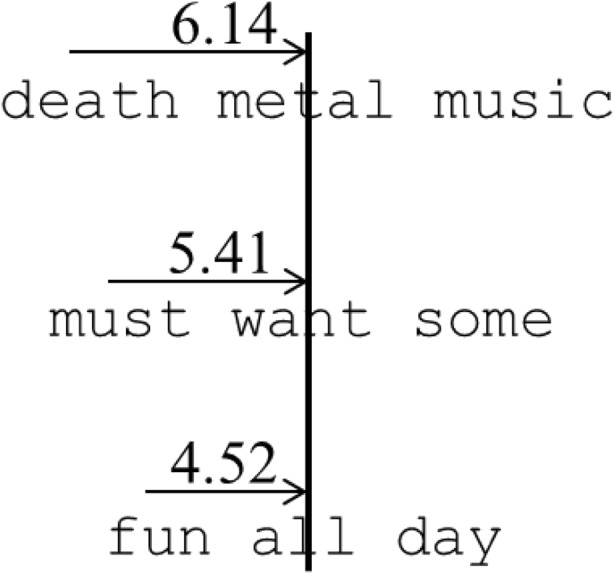
An illustration of how the preferred saccade lengths (PSLs) observed by Cutter at al. were adapted to move between the preferred viewing location of one word and the center of the next word in each different uniform sentence type. The center of each word is bisected by a straight line, and the PSL is plotted moving towards them

**Fig. 2:**
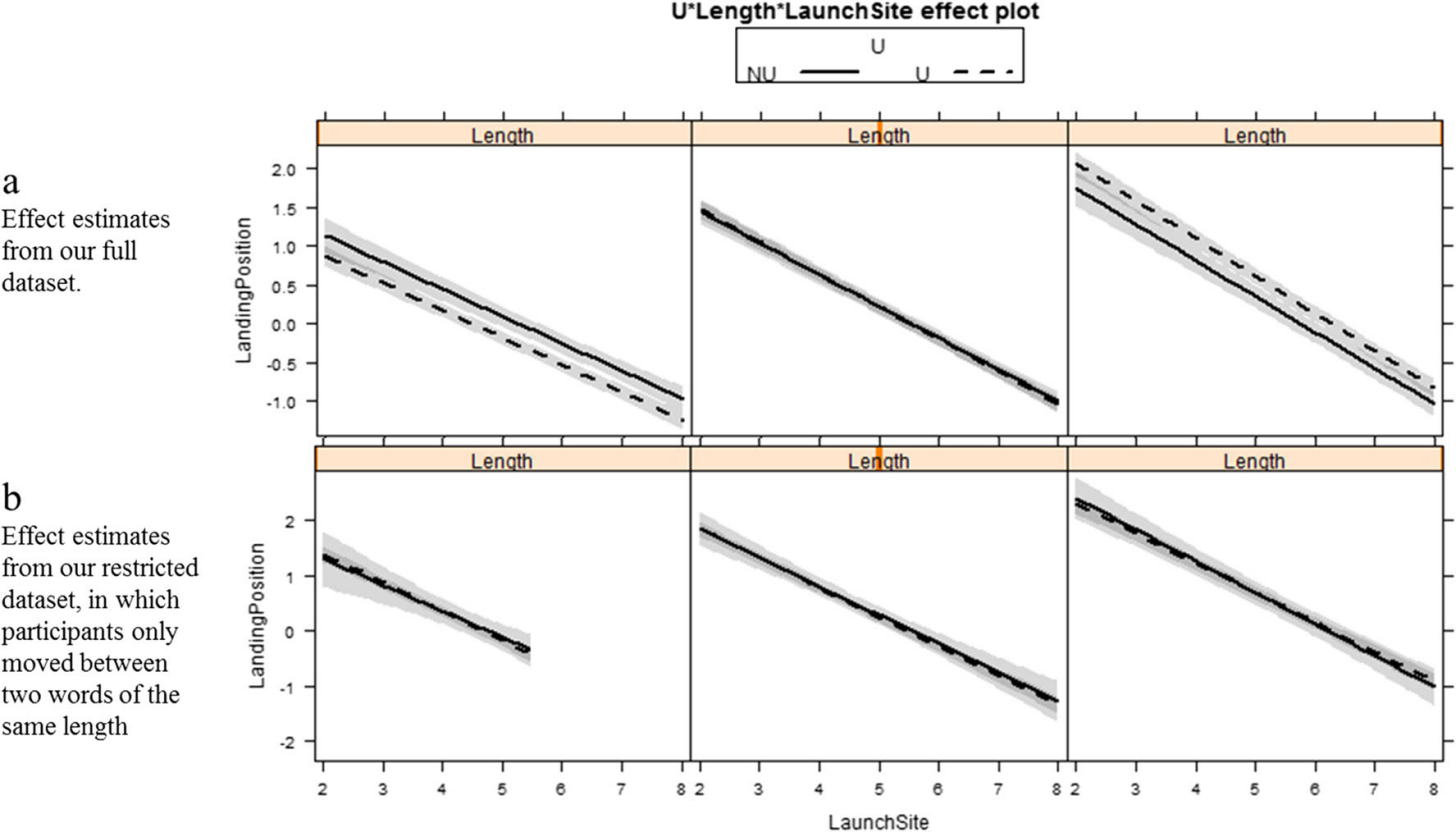
Effects estimates from the linear mixed models presented in the current paper. Initial fixation locations are estimated and plotted as a function of saccade launch site, word length, and whether a word appeared in a uniform or non-uniform sentence. The left panel plots the data for three-letter words, the middle panel for four-letter words, and the right panel for five-letter words. In (**a**) we constructed our LMM on the basis of all available data. In (**b**) our LMM was constructed on the basis of a restricted dataset in which we only examined instances of participants moving between two words of the same length and a restricted dataset. Ninety-five percent confidence bands are plotted around the model estimates

**Fig. 3:**
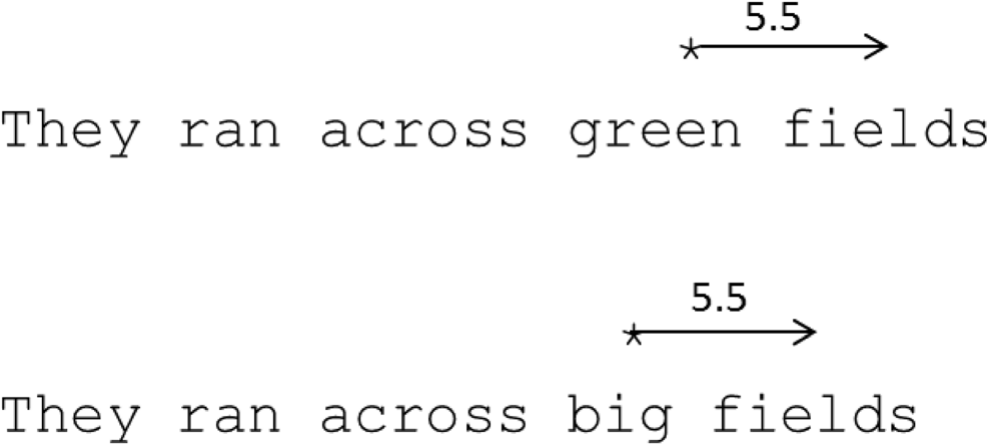
In both of the examples above a saccade is launched towards the word *fields* from 5.5 characters from its center. In a traditional experiential account of the systematic range error with a preferred saccade length (PSL), this saccade would be expected to land on the same character of *fields* regardless of the length of the launch word. In other words, according to McConkie et al.’s explanation of the systematic range error, the saccade length would only take account of the current fixation location, the center of the upcoming word, and the extent to which the difference between these deviates from a relatively fixed PSL. However, in our Centre-Based Saccade Length (CBSL) account the saccade would overshoot in the first sentence and land in the word center in the second sentence, due to the difference in length between *green* and *big*. Specifically, the CBSL in the first sentence would be 6.5 (the distance between the centers of *green* and *fields*) while in the second it would be 5.5 (the distance between the centers of *big* and *fields*). As such, the extent to which the 5.5 characters deviates from the CBSL would vary in these two different examples, leading to different levels of range error

The idea of a systematic range error has been highly influential in the field of eye movements and reading, and is implemented in the E-Z Reader (Reichle, Rayner, & Pollatsek, 2003) and SWIFT (Engbert, Nuthmann, Richter, & Kliegl, 2005) models of oculomotor control. In both models there is a fixed PSL, with 0.40 characters of error for each character of deviation from this.

Recently, Cutter, Drieghe, and Liversedge (2017) demonstrated that rather than being fixed, the PSL is malleable, adjusting to the length of words within a set of sentences. In this study participants read sentences in a blocked design in which the words were all three (e.g., *the sad boy had not had any fun all day*), four (e.g., *that tall girl near your shop must want some food*), or five letters long (e.g., *David often plays awful death metal music about Satan*), or a combination of these lengths (e.g., *Tim can often leave work about one hour early*). Cutter et al. calculated the PSL for each uniform sentence type by using linear mixed-models to determine the lauch site from which participants would land perfectly on the word center. They found that the PSL adapted during reading of each sentence type, with PSLs of 4.52, 5.41, and 6.14 characters for uniform sentences of three-, four-, and five-letter words, respectively. In contrast, when the same calculation was performed for each different word length in non-uniform sentences the PSL was 5.29, 5.63, and 5.85 characters for three-, four-, and five-letter words. In the uniform sentences these PSLs allowed readers, on average, to move from the most common landing position (and therefore the launch site of the next saccade) in one word to the center of the next (see Fig. 1). In non-uniform sentences the average word length was 3.94 letters, which might explain why the PSL in these sentences was similar to that for the uniform sentences of four-letter words, rather than the seven characters PSL observed by McConkie et al.

Cutter et al. adopted a blocked design in which participants read 30 of each sentence type in succession. As such, their effects are not problematic to the concept of a PSL; rather, they merely demonstrated that the value of the PSL adaptated in response to an accumulation of constant word length information across multiple trials. However, if such adaptation was to be shown to be very rapid, occurring on a trial-by-trial, or even word-by-word basis then this may be more problematic for the notion of a PSL. There is currently no specification in any account of how the oculomotor system might rapidly change the metrics of saccadic targeting based on a recalculation of PSL. Instead, it is assumed (implicitly at least) that the PSL is fairly fixed, being based on quite extensive experience of the length of words in the language being read. The theoretical issue at stake in the present study, then, is not just the question of the time course of PSL adaptation, but of whether the idea of a PSL (that would need to be fairly fixed) is even plausible. Thus, in the current study, we aimed to investigate whether PSL adaptation might occur on a trial-by-trial basis, or even on a word-by-word basis.

We investigated the first of these issues by presenting participants with the different sentence types used by Cutter et al. in an unblocked design, where sentences from each condition were presented in a random order. We hypothesized that if PSL adaptation occurred on a trial-by-trial basis, then we would observe similar effects to those obtained in our prior study. We would, therefore, find different PSLs for the different uniform sentence types, alongside mostly similar PSLs for the different word lengths in non-uniform sentences. We predicted similar PSLs for the different word lengths in the non-uniform condition since within these sentences participants should make saccades based on the average word length (i.e., four characters on average, plus a space between words, resulting in PSLs around five). Furthermore, we expected that participants’ saccades should land further into three-letter words from any given launch site in the non-uniform relative to uniform sentences due to the larger non-uniform sentence PSL, while the opposite pattern should be observed for the five-letter words due to the smaller non-uniform sentence PSL. We expected no effect of sentence uniformity for four-letter words. If, however, multiple blocked trials of sentences are required for PSL adaptation to occur, then Cutter et al.’s effects should not replicate. Rather, the PSL would remain largely constant across our different sentence types, with the PSL for each word length being the same for uniform and non-uniform sentences.

At this point, it is important to consider, a priori, the theoretical implications should Cutter et al.’s findings be replicated under conditions where uniform sentences are presented in a randomized sequence. Such a result would mean that adaptation in saccadic targeting can occur within a single trial and we would therefore require an alternative theoretical account to the original idea of a (fairly fixed) PSL put forward by McConkie et al. (1988). One such alternative is that the PSL may vary on a fixation-by-fixation basis, being computed on-line as a function of the length of both the word that a saccade is launched from, and the word that the saccade is targeted towards. If this was true, then the PSL on any given fixation would be equivalent to the distance between the center of the currently fixated word, and the center of the targeted word; consequently, the PSL would increase by approximately half a character for each character increase in either of these words. In our uniform sentences, the word from which a saccade was launched, and the word to which the saccade was targeted would always be the same length. As such, we would expect to see increases of a whole character for the PSL between the three-, four-, and five-letter uniform sentences. This was the pattern of effects reported by Cutter et al. Next consider the non-uniform sentences. Here there is variability in the length of the word from which a saccade was launched and the word to which the saccade was targeted. Consequently, there would be no regular and consistent difference in saccade lengths for fixations landing on words of a particular length (because the saccade extent would depend on the length of both the launch word and the target word, not just the target word). This distinction is important to note since the analysis conducted by Cutter et al. exclusively took into account variability in the length of the target word. It did not take into account the length of the word from which a saccade was launched. Furthermore, this was also the case for the analyses reported by McConkie et al. Thus, in the current experiment, if we were to obtain results consistent with those reported by Cutter et al., this would suggest that saccade metrics are computed online on the basis of the length of the word from which a saccade was launched and the length of the word to which the saccade was targeted. To be clear, this would represent a novel theoretical account of saccadic targeting in reading.

## Method

### Participants

Seventy-five students at the University of Southampton participated for course credit.

### Apparatus

Movements of the right eye were recorded using an SR Research Eyelink 1000. Sentences were presented on a single line of a ViewSonic p227f CRT monitor. The viewing distance was 78c m, with 1° of visual angle occupied by 2.81 characters of Courier font.

### Materials and design

Participants read 40 sentences of which 30 were uniform in terms of word length (i.e., three-, four- and five-letter uniform sentences) while the remaining ten were non-uniform, and constructed from a combination of three-, four-, and five-letter words. All participants viewed all sentences in a randomised order, alongside 36 filler items with a natural range of word lengths.

### Procedure

Participants were presented with a consent form and information sheet. They were seated in front of the eye-tracker and a headrest was used for stabilization. A three-point horizontal calibration was performed, with an acceptance criterion of an average error below 0.25°.

Each trial began with a drift check in the center of the monitor, followed by a drift check in the position of the center of the first word of the sentence. If either drift check indicated more than 0.3° of error the participant was recalibrated. After the drift checks a sentence appeared. Participants read for comprehension, and pressed a button after reading a sentence. The experimental sentences were preceded by six practice trials. On one-third of trials participants were presented with a yes/no comprehension question, and responded using a button box. The experiment lasted approximately 25 minutes.

## Results

Across all participants 94% of comprehension questions were answered correctly. Our dataset and the R script used to analyze it are available through the Open Science Framework (https://osf.io/kpgqb/). In total, 16,364 observations were available for analysis after data exclusion criteria (see online materials) were applied.

To determine whether our manipulation led to an adaptation in the PSL we constructed a linear mixed-effect model in which the initial landing position within a word relative to the word center was our dependent variable, while the length of word the eyes landed on, sentence uniformity, and launch site from the center of the word were treated as fixed factors. We included two- and three-way interactions between these variables. We treated each individual word and subject as random factors, with the maximal random structure that would converge. We used the lme4 (Version 1.1-7; Bates, Maechler, Bolker, and Walker, 2014) package in R (2013) to construct this model. Model output is shown in Table 1, while estimated effects are plotted in Fig. 2a.

Our model demonstrated significant main effects of all three predictor variables, and significant two-way interactions of word length with both sentence uniformity and saccade launch site. The remaining interactions were non-significant. As launch site increased participants landed nearer the beginning of a word, replicating the range error effect. This effect was larger for longer words, due to there being more potential landing positions in longer words. Most interesting was the effect of whether a word of a certain length appeared in a uniform or non-uniform sentence. This effect can clearly be seen in Fig. 2a. From any given launch site participants’ saccades landed further into a three-letter word in a non-uniform relative to uniform sentence, while their saccades landed further into a five-letter word in a uniform relative to non-uniform sentence. Participants’ saccades landed the same distance into a four-letter word from a given launch site regardless of whether it was presented in a uniform or non-uniform sentence.

The most important part of our prior study to replicate was the shift in PSL between our different uniform sentence types, alongside a relatively stable PSL for different word lengths within non-uniform sentences. To assess this we used the Effects library (Fox, Weisberg, Friendly, & Hong, 2015) to obtain estimates of the PSL from our LMM, by locating the launch site for each word length that participants switched from overshooting to undershooting the word center. This gave us estimates of 4.47, 5.51, and 6.27 (formerly 4.52, 5.41, and 6.14) for the uniform sentences of three-, four-, and five-letter words, and estimates of 5.25, 5.54, and 5.76 (formerly 5.29, 5.63, and 5.85) for these word lengths within non-uniform sentences. Thus, there was a substantial change in PSL between our different uniform sentence types, while the PSL in non-uniform sentences remained relatively constant for different word lengths. This represents a clear replication of the effects of Cutter et al. (2017).

As discussed above, we were also interested in testing an alternative account of the systematic range error, whereby the PSL for any given fixation was determined by the length of both the fixated word, and the word the saccade was targeted towards. To this end, we identified instances in our non-uniform sentences when readers made saccades between two adjacent words of identical length, a situation that was comparable to that which existed in the uniform sentences. We then repeated our original analysis (exclusively for adjacent words of the same length). To maximize comparability between the uniform and non-uniform sentences, we restricted the dataset under consideration for the uniform sentences to include only saccades between adjacent words. This dataset included 621 observations for non-uniform sentences and 7,806 observations for uniform sentences. We constructed a LMM with word length, launch site, and sentence uniformity as predictor variables and landing positon as a dependent variable (see Fig. 2b, and Table 1). From Fig. 2b it can be seen that when we controlled for the length of the launch site word in non-uniform sentences, there was no modulation by uniformity of the effect of launch site on landing position for a word of a certain length. That is, the differences we observed for three- and five-letter words in our original analysis were no longer present. Using the Effects Library we obtained estimated PSLs of 4.66, 5.48, and 6.28 for three-, four-, and five-letter words in the uniform sentences, replicating our original analysis. More interestingly, we obtained PSLs of 4.76, 5.55, and 6.22 for three-, four-, and five-letter words in non-uniform sentences, values that are extremely similar to those for the uniform sentences.^1^ Our results very clearly demonstrate that saccade metrics appear to be computed on line and are influenced by the length of the word from which a saccade is launched and the length of the word to which a saccade is targeted.

## Discussion

In the current study we set out to investigate whether the adaptation of the PSL observed by Cutter et al. (2017) occurs on a trial-by-trial basis, as opposed to requiring multiple consecutive exposures to sentences with words of the same uniform length. We presented participants with sentences that varied in their word length (three-, four-, and five- letter uniform word length sentences interspersed with non-uniform sentences) in an unblocked design. Our results indicated that adaptation occurred rapidly on a trial-by-trial basis. Additional analyses examining saccades between words of the same length embedded in non-uniform sentences showed results that were quite comparable to those observed in comparable uniform sentences, indicating further, that adaptation appeared to occur on a word by word basis. Together, these results indicate that readers do not plan and execute saccades based on a PSL metric established over a relatively extended period (i.e., an experientially based metric), but instead compute saccade metrics moment to moment on the basis of perceptual information about the length of the word under fixation and the length of the word to which a saccade is to be targeted. The present results indicate that the concept of a relatively fixed PSL as advocated by McConkie et al. is erroneous and below we provide details of an alternative, novel mechanistic account of saccade targeting in reading.

Typically, the systematic range error has been modeled as a phenomenon governed by an algorithm in which the PSL is a critical factor exerting an influence over saccadic targeting in the same way across an entire sentence. This approach has been adopted in both the E-Z Reader (Reichle, Rayner, & Pollatsek, 2003) and SWIFT (Engbert, Nuthmann, Richter, & Kliegl, 2005) models. We began the current research under the assumption that this approach was mostly correct, though we assumed that the PSL might be malleable, varying depending on the average length of words in a text, and with that average word length being gauged over a comparatively extended period of reading (e.g., multiple sentences). However, our data showed that this assumption was incorrect. It is clear from the results that saccade metrics are determined on a fixation-by-fixation basis by three things: (1) the length of the word that the eyes are fixating, (2) the length of the word to which the reader will make a saccade, and (3) given that it still demonstrated a significant effect in our second LMM, the distance between the current fixation position and the upcoming word center. That is, saccades are actually computed on the basis of the distance between the launch word center and the target word center, combined with the distance between the current fixation location and the upcoming word center (see Fig. 3). To us, then, a more accurate label for this saccade metric might be a Centre-Based Saccade Length (CBSL). It is the first two of these three factors that combine in the calculation of the CBSL, and it is the deviation between the third factor and the CBSL that leads to a range error. Next, it is important to consider how the CBSL may be calculated.

It is our contention that the CBSL may be based upon information extracted regarding the length of the word that a reader is launching a saccade from and the word towards which they are launching a saccade while these words are in the parafovea. Essentially, while readers are fixated on a word (e.g., word *n*) they are able to extract information about the length of two upcoming words in the parafovea (i.e., word *n*+1 and word *n*+2). As such, readers can estimate the length of a saccade between the center of word *n*+1 and word *n*+2 while fixated on word *n*. This estimated saccade length would represent the length of saccade that a reader would need to make between word *n*+1 and word *n+*2 in the ideal scenario that their saccade towards word *n*+1 is entirely accurate, landing directly on the center of word *n*+1. This is the CBSL. However, saccadic targeting is affected by random motor error, meaning that the landing position on word *n*+1 (and thus launch site of the saccade to word *n*+2) will not necessarily be the center of that word. Under these circumstances the CBSL does not represent the length of the saccade necessary to take the reader’s point of fixation to the center of word *n*+2 (since the launch site is some distance from the center of word *n*+1, and therefore, the CBSL does not represent the length of a saccade to the center of word *n*+2). Consequently, the reader must adjust the saccade length between these two words to either be longer or shorter than the CBSL once information about the landing position on word *n*+1 becomes available. This would require the reader to arrive at a compromise between the parafoveally-derived CBSL and the launch site based saccade. This compromise would produce the range error, whereby participants make a saccade somewhere between the length of the CBSL and the launch site based saccade length.

In terms of evidence supporting the idea that readers are able to estimate the length of word *n*+1 and word *n*+2 while still fixated on word *n*, it has been shown that readers can extract information from 14–15 characters to the right of fixation, including spacing information (McConkie & Rayner, 1975; see Cutter, Drieghe, & Liversedge, 2015, for a review). As such, from the fixated word (word *n*) readers are capable of extracting word length information about the two upcoming words (word *n*+1 and word *n*+2), so long as they are not especially long.

In closing, we set out to investigate how rapidly readers could adjust their saccade metrics, specifically questioning whether the PSL was relatively fixed and based on a fairly extended period of reading text with words of a stable average length, that is, an experiential account of saccadic targeting. Our results, instead, favored a more flexible perceptual account, whereby the system controlling saccadic targeting computes saccade targets rapidly on a fixation by fixation basis. On the basis of our findings we believe that McConkie et al.’s longstanding claims regarding range error computation in reading may not actually be correct. Instead, to us, it appears that readers make saccadic targeting decisions on line, on a fixation by fixation basis, using a Centre-Based Saccade Length estimate from parafoveal word length information combined with target distance on the following word.

## Footnotes

1 It is worth noting that a reanalysis of the data from Cutter et al. (2017), in which we only took account of saccades made between two words of the same length within the non-uniform sentences, yielded similar PSLs. In the uniform sentences there were PSLs of 4.49, 5.36, and 6.11 and in the non-uniform sentences PSLs of 4.64, 5.64, and 6.23 characters for the three, four, and five-letter words.
